# Feasibility and Acceptability of a Mindfulness-Based Smartphone App among Pregnant Women with Obesity

**DOI:** 10.3390/ijerph20075421

**Published:** 2023-04-06

**Authors:** Kerrie Ward, Anjali Herekar, Peiyi Wang, Karen L. Lindsay

**Affiliations:** 1 School of Medicine, University of California, Irvine, 1001 Health Sciences Road, Irvine, CA 92617, USA; 2 Department of Psychological Science, University of California, Irvine, 4201 Social and Behavioral Sciences Gateway, Irvine, CA 92617, USA; 3 Department of Pediatrics, School of Medicine, University of California, Irvine, 3800 Chapman Ave. Suite 2200, Orange, CA 92868, USA; 4 UCI Susan Samueli Integrative Health Institute, College of Health Sciences, 856 Health Sciences Road, Suite 4600, Irvine, CA 92617, USA

**Keywords:** mindfulness, obesity, prenatal depression, eating behaviors, pregnancy, maternal wellbeing

## Abstract

Maternal obesity is associated with an increased risk for prenatal depressive symptoms. Mindfulness-based interventions (MBIs) have been shown to reduce the risk of prenatal depression. This pilot study assesses the feasibility and acceptability of a smartphone-based MBI among pregnant women with obesity, and its potential for improving maternal mental and behavioral health outcomes. Five second-trimester pregnant women with a prepregnancy body mass index > 30 kg/m^2^ participated in a 30-day audio-guided mindfulness practice using the Headspace app. All participants engaged in the pregnancy module, while three concurrently engaged in the mindful eating module. Daily engagement with the app was tracked and a post-trial survey assessed maternal acceptability. Validated pre- and post-trial questionnaires explored changes in perceived stress, anxiety, depression, and eating habits. All participants completed the study with varying levels of adherence to the prescribed daily practice; the average number of days of engagement was 23/30 (77%) for the pregnancy module and 20/30 (67%) for the mindful eating module. All subjects reported some degree of perceived benefit, and none reported negative experiences. Trends were observed for improvements in maternal mental wellbeing and eating behaviors. This pilot study shows that a smartphone-based MBI is feasible, acceptable, and perceived to provide benefit among pregnant women with obesity.

## 1. Introduction

Maternal obesity is a major obstetric risk factor for several comorbidities affecting both mother and baby, including gestational diabetes, hypertension, and pre-eclampsia, as well as increased stillbirths, neonatal deaths, neonatal intensive care unit admissions, preterm births, and congenital abnormalities [1]. Multiple studies have also shown maternal obesity in early pregnancy to be a risk factor for prenatal and postnatal depressive symptoms [2,3]. In turn, prenatal depression is associated with poor diet quality in pregnant individuals [4]. Mindfulness-based interventions (MBIs), such as mindfulness-based stress reduction, have been shown to reduce depressive symptoms during pregnancy [5]. Additionally, MBIs have been shown to improve eating behaviors, particularly emotional eating, external eating, binge eating, and weight and shape concern, in nonpregnant cohorts [6].

Newer smartphone-based mindfulness applications now provide users with a convenient and accessible way to practice daily meditation and mindfulness exercises. These technologies differ from traditional mindfulness training practices, in that they are self-directed, mobile, and inexpensive. However, the utility and acceptability of smartphone apps as a tool for delivering MBIs has not been well studied in the pregnant population with obesity. The aim of this study is to assess the feasibility and acceptability of a smartphone-based MBI in pregnancy. Secondly, we explore the potential for eliciting changes in maternal mental health (stress, depression, anxiety, and affect) and eating behaviors through the smartphone-based mindfulness practice.

## 2. Materials and Methods

### 2.1. Participants and Procedures

Between March and June 2022, research assistants screened the appointment schedule list for prenatal clinics at the University of California, Irvine Medical Center, using electronic medical records to determine potential eligibility and obtain contact details. Prospective participants were then contacted via phone calls and emails by research assistants to provide further details about the study and to conduct a comprehensive screening and eligibility survey. Eligibility criteria required participants to be 18 years of age or older, pregnant with a singleton, intrauterine pregnancy, less than 24 weeks gestation at enrollment, have a prepregnancy body mass index (BMI) greater than 30 kg/m^2^, and have a smartphone with internet access. Given that mental wellbeing and health behaviors may fluctuate across pregnancy, less than 24 weeks gestation was chosen as a cut-point to help standardize timing in pregnancy among participants. English- or Spanish-speaking individuals were eligible. The prepregnancy BMI was computed from measured weight and height if available from the electronic medical records, or from participant self-report. Participants could not have a current diagnosis or be undergoing treatment for any psychiatric disorder, or have previously completed a formal mindfulness course. The protocol was approved by the institutional review board of the University of California, Irvine, and participants provided written informed consent at the time of enrollment during an introductory online visit (via Zoom) with a research coordinator.

Participants were enrolled in a 30-day audio-guided mindfulness practice using the Headspace app. Baseline sociodemographic, behavioral, and psychosocial questionnaires were administered to participants by research personnel prior to engagement with the app using the Research Electronic Data Capture (REDCap) platform. Participants were randomized to either complete the pregnancy module only or to simultaneously participate in the pregnancy and mindful eating modules. The original recruitment target was 10 participants, with 5 in each group. However, limited staff resources prevented us from recruiting more than five participants in total for this pilot study. No control group was selected as the primary endpoint of the study was maternal acceptability and feasibility of the app.

Each day during the 30-day intervention, the subjects were requested to participate in a 10 min guided mindfulness meditation with a focus on pregnancy wellbeing (i.e., the mindful pregnancy module) using the Headspace app. For those assigned to also complete the mindful eating module, they engaged in an additional daily 10 min mindfulness practice focused on their eating habits using the app, totaling 20 min daily participation time. The app provides prerecorded, audio-guided meditation sessions with a variety of instructor voices to choose from. However, the script is standard regardless of the instructor. Each module is 30 days in duration and structured to encourage users to engage once daily for 10 min. Weekly text message reminders were sent to participants by the study team as motivation for ongoing engagement.

Participant engagement with the app and adherence to the protocol were tracked by the research team as a measure of feasibility. For each participant, the total number of days of completed mindfulness practice for the assigned modules within the 30-day intervention period was recorded.

### 2.2. Assessments

The initial sociodemographic survey included participant age, gestational age, highest level of education, household income, number of household members, health insurance type, and ethnicity. Income-to-poverty ratios were calculated from participant-reported income and number of household members relative to the 2021 United States Census Bureau Poverty Thresholds. Pre- and postintervention assessments of psychological wellbeing and eating behaviors were conducted using standardized and validated questionnaires. Maternal depression, stress, and anxiety symptoms were measured using the Edinburgh postnatal depression scale (EPDS: maximum score 30; possible depression > 12) [7], perceived stress scale (PSS: total score range 0–40) [8], and pregnancy-related anxiety questionnaire (PRAQ: total score range 10–50) [9], respectively. Maternal affect was assessed with the positive and negative affect schedule (PANAS: positive and negative scores ranging from 10 to 50) [10]. Eating behaviors were assessed with the mindful eating questionnaire (MEQ: total score range 1–4) [11] and three-factor eating questionnaire (TFEQ: three scores: uncontrolled eating, cognitive restraint, and emotional eating with maximum scores of 16, 21, and 14, respectively) [12]. Notably, the EPDS was not used as a screening tool to determine eligibility regarding depression status, but was instead used as a tool to gather data about the level of depressive symptomatology.

At the postintervention time point, a feasibility and acceptability form collected data on participants’ experiences with the mindfulness modules and the app in general using 5-point Likert scales ranging from ‘very easy’ to ‘very difficult’, and one open-ended free-text question to gather any other comments about their experiences participating in the study. All questionnaires were completed online through REDCap during virtual meetings with a research assistant.

### 2.3. Data Analysis

Continuous variables were summarized using mean values and standard deviations. Categorical variables were summarized using counts and percentages. Due to the small sample size, a statistical analysis was not performed to assess pre–post changes in psychological or eating behavior scores, nor to determine differences in changes in each score between those who completed the pregnancy module only and pregnancy plus the mindful eating modules. However, data were described and stratified by group.

## 3. Results

A total of 136 pregnant women were flagged as potentially eligible during the initial electronic medical record screening process. Of those, 58 were successfully contacted and introduced to the study, but 9 were ineligible (pregnancy loss, fetal anomaly, and recent diagnosis of mental health conditions), 25 declined participation, and 11 were lost to follow-up after requesting more time to consider the study. Thirteen pregnant women underwent a full screening for eligibility and, of those, ten were deemed eligible, but five proceeded to participate while the other five were lost to follow-up before consenting. Thus, we found an eligibility rate of 20% and a recruitment rate of 50%.

The mean participant age was 36.4 ± 6.8 years, mean gestational age at enrollment (calculated from estimated due date) was 19 ± 4 weeks, and mean prepregnancy BMI was 34.1 ± 3.3 kg/m^2^. One of the five participants was primiparous. Four of the participants were of Hispanic ethnicity and all were English speaking. All five participants were retained at the postintervention follow-up encounter (100% retention rate).

The two participants using the pregnancy module alone had a 100% adherence rate, while the average days of pregnancy module adherence for all participants was 23/30 (77%). For the three participants who also participated in the mindful eating module, the average days of mindful eating module adherence was 20/30 (67%). Participants with <100% adherence to either module engaged with the app intermittently throughout the 30-day intervention period, missing sporadic days of practice. Table 1 describes further demographic information along with engagement data for each participant.

Results for the pre- and postintervention questionnaires are displayed in Table 2. On average, a decreasing trend from pre–postintervention was observed in scores for depressive symptoms, perceived stress, pregnancy-related anxiety, negative affect, emotional eating, and uncontrolled eating behaviors. When stratified by group, the decrease in negative affect was only apparent among those completing the pregnancy module only, while the decrease in perceived stress was only apparent among those in the pregnancy plus mindful eating module group. Additionally, we noted that those assigned to the mindful eating module reported a small increase in MEQ score versus no change among those only completing the pregnancy module.

**Figure 1:**
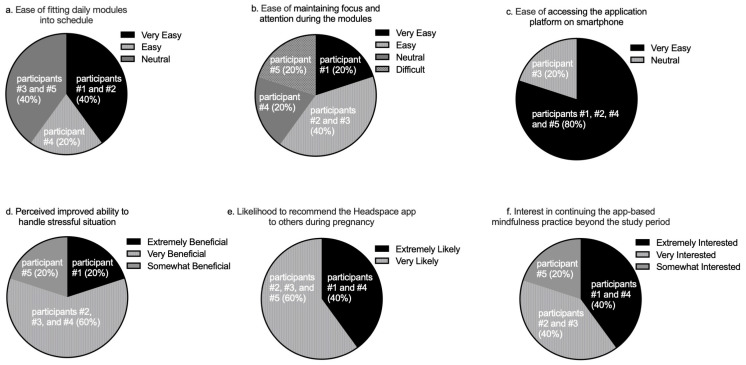
Participants’ responses regarding their experiences in participating in the study and using the Headspace app (*N* = 5). All questions were asked on a 5-point Likert scale as follows: (**a**–**c**) response options ranged from very difficult to very easy; (**d**) response options ranged from not at all beneficial to extremely beneficial; (**e**) response options ranged from extremely unlikely to extremely likely; (**f**) response options ranged from extremely uninterested to extremely interested.

Participant feedback on the study was generally positive; all subjects reported some degree of perceived benefit from the mindfulness practice, and none reported negative experiences. Figure 1 depicts participants’ responses for the following domains: their perceived ease of engaging in the daily practice (Figure 1a,b) and utilizing the Headspace application (Figure 1c), their perceived benefit in bringing awareness to or handling stressful situations as a result of the training in the pregnancy module (Figure 1d), their likelihood of recommending the Headspace app as a mindfulness tool to other pregnant women (Figure 1e), and their interests in continuing an app-based mindfulness practice in pregnancy (Figure 1f). We noted that one participant reported that maintaining focus and attention during the daily mindfulness practice was difficult (Figure 1b). Furthermore, that same participant was only somewhat interested in continuing the daily mindfulness practice beyond the study period (Figure 1f). In contrast, the remaining participants reported greater ease in maintaining focus and attention during the daily practice and selected either ‘very interested’ or ‘extremely interested’ as a response for continuing the app-based mindfulness practice.

Additionally, free-text feedback quotes about participants’ experiences of the study are listed below:

- Participant 1: “Headspace help me a lot on my every day. Like going to work with a better mood and more happy”.
- Participant 2: “I found benefits in terms of being able to keep my nighttime routine before sleep, better quality sleep, less irritability during the day as well”.
- Participant 3: “I was not able to download the app, but I was able to log in through their website”.
- Participant 4: “I’m glad I took part of this headspace app study. I enjoyed it and it taught me how to take time to relax and breathe. I especially learned a lot from the mindfulness eating modules. I learned to pay more attention to what I eat and if I’m truly hungry or eating because of emotions”.
- Participant 5: “It was calming and relaxing, but some of the messages were not very helpful and possibly harmful (mostly the eating one). I found the hyper focus on what you are eating being ‘in the moment’ one harmful because overly focusing on my food made me stop eating and uninterested in my food even though I wasn’t full yet. Others were very repetitive, and I did find it comical that I had a man teaching me about my pregnancy (I picked him over the others because I was used to his voice.). It felt a bit man-splaining at times. I had considered switching to a female coach about halfway through, but didn’t know if I would lose my progress and I didn’t want to risk it”.

The participants’ written feedback illustrated several positive outcomes, specifically regarding the pregnancy module on the app. One stated that because of this practice, she experienced elevated mood compared to her baseline. Another participant described mood improvements as a result of better sleep quality, noting an improved routine prior to sleep, which she attributed to the mindfulness practice. Another perceived benefit was relaxation during the practice. Two participants stated that the mindfulness modules served as a relaxation technique that was calming to them during their pregnancy.

There were variable responses to the mindful eating modules. One participant particularly gained insight from these modules, stating she learned how to observe how her emotions were influencing her eating patterns. However, another participant found the mindful eating module to be somewhat unhelpful due to overly focusing on the process of eating, which negatively impacted her appetite.

## 4. Discussion

This paper highlighted the feasibility and acceptability of a smartphone-based mindfulness practice in pregnant individuals with obesity. All participants completed the study and had at least 50% adherence to the prescribed daily practice, with each participant reporting some benefit from the intervention. The two participants who were assigned only the pregnancy modules adhered to all 30 days of the prescribed practice. Of note, the two participants who had approximately 50% adherence to the 30-day mindfulness in pregnancy module, were two of the three participants assigned to the additional mindful eating module. Assigning one module daily may be more attainable as to not overwhelm participants with larger time commitments.

Two previous studies assessed the use of smartphone-based mindfulness practices in pregnancy. Kubo et al. demonstrated feasibility and acceptability of a mobile mindfulness practice, specifically for pregnant individuals with moderate to moderately severe antenatal depressive symptoms. Compliance in that study was recorded, as over half of the participants used the app more than 50% of the days of the week during the six-week intervention [13]. Additionally, Green et al. found that 52% of pregnant participants engaged with a mobile app meditation guide three times or more per week throughout their pregnancy [14] and reported the intervention to be helpful for sleep, anxiety, and stress. Interestingly, participants of that study requested more pregnancy-specific content. All participants in our study were assigned to complete the pregnancy mindfulness module on the app. The high compliance rates and absence of negative feedback reported from this module supported the feasibility of using the Headspace app to deliver pregnancy-tailored content for MBIs in future studies.

Our study demonstrates the overall acceptability of a smartphone app to deliver an MBI in pregnancy, as all participants reported some level of benefit from participation, and most participants desired to continue using the app beyond the study period. Only one participant reported difficulty in maintaining focus and attention while engaging in the daily mindfulness practice, which possibly contributed to a lower desire compared to the other participants to continue using the app after the 30-day study period was completed. For future studies utilizing apps to deliver an MBI, this may indicate a need for personal follow-up with those who struggle to engage in the mindfulness practices to provide support and encouragement in an effort to reduce attrition rates.

The feedback from participants reflects the benefits of the mindfulness-based app in improving psychological wellbeing—a result that has been observed in nonpregnancy populations using mindfulness meditation applications [15,16]. However, one participant reported negative feedback regarding her experience of the mindful eating module. Based on this feedback, it should be noted that some participants may be more prone to dysfunctional eating patterns than others. It is important to consider that a self-directed mindful eating practice for beginners may not always be appropriate depending on their ideas and relationship with food. A prior study among nonpregnant individuals utilizing an alternative mindfulness-based smartphone app similarly reported negative feedback among a minority of participants, although the practice in that study was not specific to mindful eating [17]. While mindfulness practices have been shown to support healthy eating behaviors in individuals with eating disorders [18], these individuals may be better suited to a guided practice with a trained mindfulness instructor as well as medical treatment if there is a clinical eating disorder. It may be prudent for future studies that utilize an app-guided mindful eating practice to screen out those with prior history of or current disordered eating.

This was the first pilot study to test the acceptability of the Headspace app in a cohort of pregnant women with obesity and without psychiatric conditions. Notably, we tested the impact of mindfulness training on eating behaviors in addition to psychological outcomes. Of note, four of five participants were of Hispanic ethnicity, a group that is typically more socially disadvantaged and rarely engaged in mindfulness-based research [19]. This population has also been shown to have higher rates of obesity in the United States than those who are non-Hispanic [20]. It remains to be determined if engaging this group in an accessible, remotely delivered mindfulness practice could potentially support improvements in health behaviors to mitigate the risks associated with prenatal obesity.

This study was limited by its small sample size. Despite this, trend improvements in maternal mental wellbeing and eating behavior were observed. Testing for statistical significance in changes in psychological and eating behavior scores was deemed unreliable, particularly when assessing compliance and comparing outcomes between the group that participated in the pregnancy module alone against the group participating in the additional mindful eating module. Additionally, there was no comparison group of pregnant women not using the app to evaluate if the observed trends in depression scores and eating behaviors were attributed to the mindfulness practice or other factors over the course of the month. There was also a risk of participation bias, as those individuals who were more interested in mindfulness practices may have been more likely to participate in the study.

## 5. Conclusions

This pilot study highlighted the feasibility and acceptability of a smartphone-based mindfulness practice in pregnant individuals, particularly when using a module tailored to mindfulness surrounding pregnancy. Future controlled studies in larger cohorts are required to assess the impact of this type of MBI on maternal psychological wellbeing and eating behaviors. Further data would help determine if app-based mindfulness is an appropriate and scalable intervention to support the wellbeing of pregnant individuals and help identify subgroups who may benefit most from this practice, ultimately, improving maternal and fetal outcomes.

## Figures and Tables

**Table 1:** Demographics description and engagement data for each participant.

Participant Number	Participant Age	Gestational Age	Education Level	Marital Status	Income: Poverty Ratio	Health Insurance Type	Ethnicity	Modules Assigned	Days of Pregnancy Module Completed	Days of Mindful Eating Module Completed
1	33.3	16w3d	High School or GED	Married	1.0	Government Insurance	Of Hispanic, Latino, or Spanish Origin	Pregnancy Module Only	30	N/A
2	32.7	23w4d	Graduate Degree	Never Married, Single	14.5	Commercial Health Insurance	Of Hispanic, Latino, or Spanish Origin	Pregnancy Module Only	30	N/A
3	48.6	12w6d	Bachelor’s Degree	Married	1.6	Government Insurance	Of Hispanic, Latino, or Spanish Origin	Pregnancy + Mindful Eating Modules	15	17
4	32.7	20w3d	Associate Degree	Married	8.1	Commercial Health Insurance	Of Hispanic, Latino, or Spanish Origin	Pregnancy + Mindful Eating Modules	28	28
5	34.7	23w2d	Bachelor’s Degree	Married	5.0	Commercial Health Insurance	Not of Hispanic, Latino, Or Spanish Origin	Pregnancy + Mindful Eating Modules	16	16

Income: poverty ratio describes the total family income divided by the poverty threshold relevant to the household size. Government insurance includes federal, state, or local plans. Commercial insurance includes private, health maintenance organization, or preferred provider organization plans. GED, general educational development test.

**Table 2:** Pre- and postintervention questionnaire scores for all participants and stratified by modules assigned.

Questionnaire Name and Subscales	All Participants (n = 5)		Pregnancy Modules Only (n = 2)		Pregnancy + Mindful Eating Modules (n = 3)	
	Preintervention (Mean ± SD)	Postintervention (Mean ± SD)	Preintervention (Mean ± SD)	Postintervention (Mean ± SD)	Preintervention (Mean ± SD)	Postintervention (Mean ± SD)
Edinburgh Postnatal Depression Scale	9.0 ± 4.3	5.2 ± 3.1	7.5 ± 7.8	2.5 ± 3.5	10.0 ± 1.7	7.0 ± 1.0
Perceived Stress Scale	16.6 ± 7.5	13.2 ± 3.4	12.0 ± 11.3	12.5 ± 4.9	19.7 ± 3.5	13.7 ± 3.2
Pregnancy- Related Anxiety Questionnaire	24.2 ± 7.5	20.0 ± 6.4	18.5 ± 3.5	13.5 ± 2.1	28.0 ± 7.2	24.3 ± 3.2
Positive and Negative Affect Schedule						
Positive Affect	34.2 ± 4.4	37.4 ± 5.6	36.5 ± 6.4	41.0 ± 7.1	32.7 ± 3.1	35.0 ± 4.0
Negative Affect	19.2 ± 3.0	15.6 ± 4.2	20.0 ± 1.4	11.5 ± 2.1	18.7 ± 4.0	18.3 ± 2.1
Mindful Eating Questionnaire	2.9 ± 0.2	3.1 ± 0.2	2.9 ± 0.2	2.9 ± 0.1	2.9 ± 0.3	3.2 ± 0.1
Three Factor Eating Questionnaire						
Uncontrolled Eating	18.8 ± 5.9	14.4 ± 3.4	14.0 ± 7.1	13.0 ± 4.2	22.0 ± 2.6	15.3 ± 3.2
Cognitive Restraint	14.0 ± 3.9	16.2 ± 3.4	12.0 ± 4.2	19.0 ± 0.0	15.3 ± 3.8	14.3 ± 3.2
Emotional Eating	13.8 ± 4.4	10.4 ± 2.4	10.5 ± 6.4	9.0 ± 2.8	16.0 ± 1.0	11.3 ± 2.1

## Data Availability

The data presented in this study are available in the Supplementary Materials.

## Supplementary Materials

The following supporting information can be downloaded at: https://www.mdpi.com/article/10.3390/ijerph20075421/s1, Dataset S1: Demographic and survey data of all participants.
